# Hypertensive retinopathy in pre‐eclampsia and its association with disease severity and neonatal outcomes: A retrospective cohort study

**DOI:** 10.1002/ijgo.70818

**Published:** 2026-01-22

**Authors:** Gabriele Saccone, Francesco Matarazzo, Mariarosaria Motta, Marika Rovetto, Michele Rinaldi, Maurizio Guida, Ciro Costagliola

**Affiliations:** ^1^ Department of Neuroscience, Reproductive Sciences and Dentistry, School of Medicine University of Naples Federico II Naples Italy; ^2^ Department of Physics University of Naples Federico II Naples Italy

**Keywords:** eclampsia, hypertensive retinopathy, intensive care units, neonatal, pre‐eclampsia, premature birth, retina

## Abstract

**Introduction:**

Pre‐eclampsia (PE) involves systemic endothelial dysfunction and microvascular injury, yet routine obstetric care lacks noninvasive readouts of maternal microvascular health. We evaluated whether hypertensive retinopathy (HR) detected during pregnancy is associated with maternal disease severity and adverse neonatal outcomes.

**Methods:**

We performed a retrospective cohort study of singleton pregnancies with PE and at least one ophthalmic assessment during pregnancy. Retinal findings were graded as none, mild, moderate, or severe. Primary outcomes were maternal composite adverse outcome (severe features, HELLP syndrome, eclampsia, admission to intensive care unit) and neonatal composite adverse outcome (indicated delivery <34 weeks, small for gestational below the third percentile, admission to neonatal intensive care unit). Multivariable models adjusted for maternal confounders were performed.

**Results:**

Of 584 patients with PE with analyzable data, 182 (31.2%) had any HR (mild 20.4%, moderate 9.8%, severe 1.0%). HR was independently associated with maternal composite adverse outcome (adjusted odds ratio [aOR], 2.21 [95% CI, 1.45–3.36]) and neonatal composite adverse outcome (aOR, 2.40 [95% confidence interval (CI), 1.60–3.60]). HR was linked to earlier delivery (adjusted mean difference, −1.17 weeks) and lower birthweight *z* score (adjusted *β*, −0.34). Each one‐grade increase in HR was associated with higher odds of both primary outcomes (maternal composite outcome aOR, 1.45 [95% CI, 1.10–1.90]; neonatal composite outcome aOR, 1.53 [95% CI, 1.17–1.99]).

**Conclusions:**

In women with PE, HR is common and independently associated with maternal and neonatal adverse outcomes.

## 1 INTRODUCTION

Pre‐eclampsia (PE) remains a leading cause of maternal and perinatal morbidity and mortality.^1^ Despite standardized antenatal surveillance and evidence‐based strategies, its onset is often abrupt, its clinical course heterogeneous, and its downstream effects on the fetus substantial. A unifying pathophysiologic feature of PE is widespread maternal endothelial dysfunction with associated microvascular injury. This endotheliopathy alters vascular tone, permeability, and autoregulatory capacity among multiple organ systems and is accompanied by an antiangiogenic milieu.^2^ Reliable, noninvasive readouts of maternal microvascular health could therefore improve risk stratification and inform timing of delivery, yet such tools are limited in routine obstetric practice.

The retina offers a uniquely accessible window to the systemic microcirculation.^4^ Retinal arterioles and venules share caliber, autoregulatory mechanisms, and vulnerability to endothelial injury with other resistance vessels implicated in hypertensive disease. For decades, qualitative funduscopic signs, arteriolar narrowing, arteriovenous nicking, cotton‐wool spots, and retinal hemorrhages have been recognized in severe hypertension. In pregnancy, similar signs have been described in association with PE. However, the obstetric literature is fragmented. Many reports are small case series and standardized grading is inconsistently applied.^4^, ^5^, ^6^, ^7^, ^8^

Two gaps are especially pertinent to contemporary care. First, most prior studies have treated ocular findings as confirmatory evidence of severe hypertension rather than as potential biomarkers^8^; consequently, whether the presence and severity of hypertensive retinopathy (HR) correlate with obstetric indicators of disease severity has not been systematically quantified in representative cohorts. Second, the prognostic value of retinal findings for neonatal outcomes has rarely been explored.^5^, ^6^

### 1.1 Objective

The primary objective of this study was to determine, among pregnant individuals with PE, whether the presence and severity of HR are associated with maternal disease severity and with adverse neonatal outcomes.

## 2 METHODS

### 2.1 Study design and setting

We conducted a retrospective cohort study at University of Naples Federico II. The study period spanned January 1, 2015, to January 31, 2025.

### 2.2 Participants

#### 2.2.1 Inclusion criteria

- Singleton pregnancy.
- Diagnosis of PE.
- At least one documented ophthalmic assessment during pregnancy (nonmydriatic fundus examination and/or dilated fundus examination with a contemporaneous clinical report).

For descriptive purposes only, we also identified a normotensive control group of pregnant individuals with at least one ophthalmic assessment during pregnancy and no hypertensive disorder of pregnancy.

#### 2.2.2 Exclusion criteria

- Preexisting ocular disease likely to confound retinal assessment (e.g. proliferative diabetic retinopathy, advanced glaucoma, active uveitis, high myopia, macular degeneration).
- Pregestational diabetes with documented proliferative retinopathy.
- Multiple gestation.
- Inadequate or missing ophthalmology reports that did not allow classification of HR.

#### 2.2.3 Definitions and outcomes

Hypertensive disorders and severity PE and other hypertensive disorder of pregnancy were defined according to American College of Obstetricians and Gynecologists (ACOG) criteria^9^: new‐onset hypertension ≥20 weeks of gestation plus proteinuria or end‐organ dysfunction. Severe features included any of the following: systolic blood pressure ≥160 mm Hg or diastolic ≥110 mm Hg, thrombocytopenia (<100 × 10^9^/L), serum creatinine >1.1 mg/dL or doubling from baseline, elevated transaminases (≥2× upper limit of normal) with clinical symptoms, pulmonary edema, new‐onset cerebral/visual symptoms, or eclampsia. Early‐onset PE was defined as diagnosis before 34 weeks.

Small for gestational age (SGA) was defined as birthweight <10th percentile for gestational age, and severe SGA was defined as below the third percentile.

Ophthalmic assessments were performed as part of routine clinical care or at the obstetrician's discretion, generally after the clinical diagnosis of PE. Retinal signs were classified using the HR scale, recorded at the eye level, and summarized at the patient level as the worse‐eye grade (Tables S1 and S2)^10^:

- None: no hypertensive retinal signs.
- Mild: generalized/focal arteriolar narrowing and/or arteriovenous nicking.
- Moderate: hemorrhages and/or cotton‐wool spots and/or hard exudates.
- Severe: optic disc edema (papilledema) with or without other signs.

The primary outcomes of the study were:

- Maternal composite adverse outcome, i.e. composite of any severe features, HELLP syndrome, eclampsia, or maternal admission to intensive care unit.
- Neonatal composite adverse outcome, i.e. any of the following: medically indicated delivery before 34 weeks, severe SGA, or admission to the neonatal intensive care unit (NICU).

The secondary outcomes of the study were:

- Individual components of the above composites.
- Gestational age at delivery.
- Birthweight *z* score.
- Associations between retinopathy grade and obstetric Doppler indices (uterine artery pulsatility index, cerebroplacental ratio), performed in the third trimester of pregnancy.

### 2.3 Statistical analysis

We reported baseline characteristics as mean (SD) or median (interquartile range) for continuous variables and number (percentages) for categorical variables, and compared groups using *t* tests/analysis of variance or Mann–Whitney tests and Fisher exact tests, as appropriate. For the primary analyses, we modeled the association between HR (any vs. none) and each primary outcome using multivariable logistic regression, reporting adjusted odds ratios (aORs) with 95% confidence intervals (CIs). To evaluate dose–response, we fit logistic regression models with HR grade (none, mild, moderate, severe) entered as a continuous ordinal predictor, estimating the odds of maternal and neonatal composite outcomes per one‐grade increase. For secondary outcomes, we used linear regression for gestational age at delivery and for birthweight *z* score.

All tests were two‐sided with *α* = 0.05. Statistical analysis was performed using SPSS, version 29 (IBM).

## 3 RESULTS

### 3.1 Cohort selection and baseline characteristics

During the study period, 629 singleton pregnancies with a diagnosis of PE had at least one ophthalmic assessment during pregnancy. After excluding patients with confounding ocular disease, the final PE analysis cohort comprised 584 patients. A contemporaneous normotensive comparison group of 538 singletons was identified for descriptive purposes.

Within the PE cohort, 182 of 584 (31.2%) patients exhibited any HR (worse‐eye grade ≥mild), with distribution by grade: mild, 119 (20.4%); moderate, 57 (9.8%); and severe, 6 (1.0%). In the normotensive group, 38 of 538 (7.1%) patients showed any HR (exclusively mild).

Table 1 summarizes baseline characteristics of the PE cohort stratified by presence of HR. Compared with patients with PE without HR, those with HR had higher mean arterial pressure at ophthalmic assessment, were more often early‐onset (<34 weeks), and had less favorable Doppler indices.

**TABLE 1:** Baseline characteristics of the PE cohort, by HR status (worse‐eye grade).

Characteristic	No HR (*n* = 402)	Any HR (*n* = 182)	*P* value
Maternal age, mean (SD), years	30.8 (5.3)	31.9 (5.4)	**0.018**
Prepregnancy BMI, mean (SD), kg/m^2^	26.8 (4.7)	26.6 (5.0)	0.55
Nulliparous, *n* (%)	260 (64.7)	124 (68.1)	0.47
Chronic hypertension, *n* (%)	47 (11.7)	35 (19.2)	**0.02**
Gestational diabetes, *n* (%)	51 (12.7)	42 (23.1)	**0.002**
Pregestational diabetes, *n* (%)	24 (6.0)	9 (4.9)	0.762
Aspirin prophylaxis, *n* (%)	212 (52.7)	94 (51.6)	0.877
Mean arterial pressure at ophthalmic assessment, mean (SD), mm Hg	107 (11)	112 (12)	**<0.001**
GA at ophthalmic assessment, mean (SD), week	32.6 (3.6)	31.5 (3.9)	**0.04**
Early‐onset PE (<34 weeks), *n* (%)	98 (24.4)	80 (44.0)	**<0.001**
Uterine artery PI (subset), mean (SD)	1.32 (0.31)	1.41 (0.40)	**0.022**
Cerebroplacental ratio (subset), mean (SD)	1.23 (0.27)	1.12 (0.29)	**0.001**

*Note*: Boldface data indicates statistical significance.

Abbreviations: BMI, body mass index (BMI, calculated as weight in kilograms divided by the square of height in meters); GA, gestational age; HR, hypertensive retinopathy; PE, pre‐eclampsia; PI, pulsatility index; SD, standard deviation.

### 3.2 Primary outcomes

Among patients with PE with any HR, the maternal severe‐disease composite occurred in 107 of 182 (58.8%) patients versus 141 of 402 (35.1%) patients without HR (crude OR, 2.64). The adverse neonatal composite occurred in 99 of 182 (54.4%) patients versus 122 of 402 (30.3%) patients (crude OR, 2.74) (Table 2).

**TABLE 2:** Association between HR and outcomes in the PE cohort.

Outcome	Crude OR/*β* (95% CI)	Adjusted OR/*β* (95% CI)^a^	*P* value
Maternal severe—disease composite	2.64 (1.84–3.78)	2.21 (1.45–3.36)	**<0.001**
Adverse neonatal composite	2.74 (1.98–3.93)	2.40 (1.60–3.60)	**<0.001**
Delivery <34 weeks (indicated)	2.49 (1.63–3.80)	1.66 (1.01–2.74)	**0.04**
Severe SGA (<3rd percentile)	2.34 (1.41–3.78)	1.20 (0.62–2.31)	0.594
NICU admission	2.29 (1.55–3.39)	1.74 (1.12–2.70)	**0.014**
GA at delivery (mean difference), week	−1.16 (−1.62 to −0.70)	−1.17 (−1.65 to −0.69)	**<0.001**
Birthweight *z* score (mean difference)	−0.29 (−0.44 to −0.13)		**<0.001**
Dose–response per grade (maternal composite)	N/A	*β* −0.34 (−0.50 to −0.18)	**<0.001**
Dose–response per grade (neonatal composite)	N/A		**<0.001**

*Note*: Boldface data indicate statistical significance.

Abbreviations: BMI, body mass index; CI, confidence interval; GA, gestational age; HR, hypertensive retinopathy; N/A, not applicable; NICU, neonatal intensive care unit; OR, odds ratio; SGA, small for gestational age.

^a^ Adjusted for maternal age, BMI, nulliparity, smoking, ethnicity, chronic hypertension, pregestational diabetes, gestational diabetes, aspirin, antihypertensive class, mean arterial pressure, and gestational age at ophthalmic assessment.

In multivariable models, any retinopathy remained independently associated with both primary outcomes:

- Maternal composite adverse outcome: aOR, 2.21 (95% CI, 1.45–3.36).
- Neonatal composite adverse outcome: aOR, 2.40 (95% CI, 1.60–3.60).

In subgroup analyses by timing of PE onset, the association between HR and the adverse neonatal composite was not estimable in early‐onset PE because all neonates had the outcome, whereas in late‐onset PE, HR remained significantly associated with adverse neonatal outcome (aOR, 2.63 [95% CI, 1.31–5.29]; Table S2).

### 3.3 Secondary outcomes

Presence of retinopathy was associated with earlier delivery and less favorable neonatal indices. Mean gestational age at delivery was significantly lower with retinopathy compared with those without retinopathy (adjusted mean difference, −1.17 weeks [95% CI, −1.65 to −0.69]). Birthweight *z* score was also lower (adjusted *β*, −0.34 [95% CI, −0.50 to −0.18]).

Component outcomes showed consistent patterns:

- Medically indicated delivery <34 weeks: aOR, 1.66 (95% CI, 1.01–2.78).
- NICU admission: aOR, 1.74 (95% CI, 1.12–2.70).

In dose–response analyses using ordinal retinopathy grade (none, mild, moderate, severe), each one‐category increase was associated with higher odds of neonatal composite adverse outcome (aOR, 1.53 per grade [95% CI, 1.17–1.99]) and of maternal composite adverse outcome (aOR, 1.45 per grade [95% CI, 1.10–1.90]).

HR was associated with a trend toward higher uterine artery pulsatility index (adjusted *β*, +0.08 [95% CI, −0.01 to +0.17]; *P* = 0.07) and with significantly lower cerebroplacental ratio (adjusted *β*, −0.13 [95% CI, −0.20 to −0.06]; *P* < 0.001).

### 3.4 Sensitivity and subgroup analyses

Results were consistent across sensitivity analyses, including exclusion of women with chronic hypertension and pregestational diabetes. In subgroup analyses by PE onset, the association with the adverse neonatal composite was not estimable in early‐onset PE because all neonates had the outcome, whereas in late‐onset PE, HR remained significantly associated with adverse neonatal outcome (aOR, 2.63 [95% CI, 1.31–5.29]; Table S2). In the normotensive comparison group, the prevalence of any retinopathy was low (7.1%), and retinopathy was not associated with adverse neonatal outcomes after adjustment (aOR, 1.12 [95% CI, 0.62–2.03]).

## 4 DISCUSSION

### 4.1 Main findings

In this retrospective cohort of 584 singleton pregnancies with PE, HR was common when an ophthalmic assessment was performed during pregnancy. HR was independently associated with both the maternal severe disease composite and the adverse neonatal composite after adjustment for confounders. HR was also associated with earlier delivery and lower birthweight *z* score.

This study has several limitations, including its retrospective, nonrandomized study design. Because we relied solely on clinical ophthalmology reports, without photographic confirmation or standardized regrading, exposure classification may be subject to underascertainment and nondifferential misclassification of HR severity, likely biasing estimates toward the null. Ophthalmic assessments were clinically indicated rather than universal, introducing potential selection bias, e.g. women with more severe disease may have been more likely to be examined. Although we adjusted for key confounders, residual confounding is possible. Timing of the ophthalmic assessment varied, which may have influenced the detection of retinal signs. Important data such as serial estimated fetal weight measurements were not consistently available in the cohort. Gestational age at diagnosis was not available for all patients, therefore we adjusted for gestational age at the time of the ophthalmic assessment. Adjustment for confounders was limited by the retrospective nature of the study and incomplete documentation of exact timing. Comorbidities other than PE could not be systematically identified, limiting the ability to distinguish between isolated PE and PE with additional maternal disease. Maternal adverse outcomes were too infrequent within stratified subgroups to allow reliable subgroup analysis, limiting the evaluation of effect modification for maternal end points. In the normotensive control group, comorbidity data were incomplete, preventing attribution of the mild retinal findings to specific maternal condition. Finally, Doppler data were not available for all the included women.

### 4.2 Implication

Prior reports, typically small, single‐center series, have described HR in PE and qualitatively linked its presence to more severe maternal disease.^3^, ^4^, ^5^, ^6^, ^7^, ^8^, ^10^, ^11^ Few studies have quantified graded associations with maternal and neonatal outcomes.^8^, ^12^, ^13^, ^14^ Our cohort adds to this literature by estimating a prevalence of HR approximately one‐third among patients with PE who undergo ophthalmic assessment, demonstrating dose–response relationships across ordered HR grades, and showing independent associations with clinically relevant neonatal end points.

Our findings support the concept that retinal signs mirror maternal microvascular compromise in PE and are independently associated with adverse neonatal outcomes.^15^ From a clinical perspective, ophthalmic examination in women with PE could help identify those at heightened risk for early delivery and neonatal adverse outcomes.^16^ However, before advocating broader implementation, prospective and multicenter studies are required to define thresholds, workflow, and cost‐effectiveness.

## 5 CONCLUSIONS

In our cohort, HR was frequent and independently associated with maternal disease severity and adverse neonatal outcomes. These findings support closer obstetrics‐ophthalmology collaboration and motivate prospective studies to test whether retinal‐informed care pathways can improve perinatal outcomes.

## AUTHOR CONTRIBUTIONS

Gabriele Saccone conceived and designed the study, supervised data collection, performed statistical analyses, and drafted the manuscript. Francesco Matarazzo contributed to data analysis and interpretation. Mariarosaria Motta, Marika Rovetto, and Michele Rinaldi contributed to data acquisition and clinical input. Maurizio Guida and Ciro Costagliola provided critical revision of the manuscript for important intellectual content. All authors approved the final version and agree to be accountable for all aspects of the work.

## CONFLICT OF INTEREST STATEMENT

The authors declare no conflicts of interest.

## ETHICS STATEMENT

The study was approved by the local institutional review board.

## INFORMED CONSENT

Patient consent was waived because of the retrospective design of the study and anonymization of data.

## Supporting information


Tables S1–S2

## Data Availability

The data that support the findings of this study are available from the corresponding author upon reasonable request.
