# Supplementary material for: Hypertensive retinopathy in pre‐eclampsia and its association with disease severity and neonatal outcomes: A retrospective cohort study
Source: Int J Gynaecol Obstet. 2026 Jan 22;174(1):229–34. doi: 10.1002/ijgo.70818 (PMC13278649; doi:10.1002/ijgo.70818)
Supplement: Supplementary file 1 — Tables S1–S2 [file IJGO-174-229-s001.docx]

**SUPPLEMENTARY TABLES**

**Table S1.** Simplified hypertensive retinopathy grading rubric used in this study

| **Grade** | **Definition / Findings** |
| --- | --- |
| **None** | No hypertensive retinal signs. |
| **Mild** | Generalized/focal arteriolar narrowing and/or arteriovenous nicking. |
| **Moderate** | Retinal hemorrhages and/or cotton-wool spots and/or hard exudates. |
| **Severe** | Optic disc edema (papilledema) with or without other signs. |

*Clinical descriptors recorded by ophthalmologists during routine care were mapped to four severity categories (none, mild, moderate, severe)*

**Table S2.** Association of hypertensive retinopathy with adverse neonatal composite by preeclampsia onset (early <34 weeks vs late ≥ 34 weeks)

| **Subgroup** | **Adjusted OR (95% CI)** | **Interaction p-value** |
| --- | --- | --- |
| Early onset PE (<34 weeks) | Not estimable* | Not applicable* |
| Late onset PE (≥34 weeks) | **2.63 (1.31 to 5.29)** | Not applicable* |

*PE, preeclampsia; OR, odds ratio; CI, confidence interval*

**In early-onset PE, all neonates had the adverse composite outcome (100%), leading to complete separation and non-estimable OR*

*Bold face data, statistically significant*
